# Personality traits and risk of eating disorders among Polish women: the moderating role of self-esteem

**DOI:** 10.1192/j.eurpsy.2025.1404

**Published:** 2025-08-26

**Authors:** K. Rachubińska, D. Schneider-Matyka, A. M. Cybulska, M. Nowak, E. Grochans

**Affiliations:** 1 Department of Nursing, Pomeranian Medical University, Szczecin, Poland

## Abstract

**Introduction:**

Personality dimensions should be taken into account when diagnosing individuals with disordered eating behaviours in the hope of better understanding their etiology and symptom progression and when planning treatment.

**Objectives:**

The objective of this study was to attempt to determine the moderating role of self-esteem in the relationships between personality traits included in the Big Five model among Polish women and the occurrence of eating disorders.

**Methods:**

The study was conducted among 556 Polish women from Zachodniopomorskie Voivodeship. A diagnostic survey was used as the research method, and the empirical data were collected using the following research tools: The NEO Five-Factor Inventory (NEO-FFI), Rosenberg Self-Esteem Scale (SES), ORTO – 15 *Questionnaire*, The Three-Factor Eating Questionnaire (TFEQ-13), and the authors’ original questionnaire.

**Results:**

Only the personality trait of neuroticism exhibits a statistically significant effect on the “Cognitive Restraint of Eating”, “Uncontrolled Eating”, and “Emotional Eating” scores (p<0.001). The moderation effect was demonstrated between self-esteem and the personality trait of conscientiousness on the “Cognitive Restraint of Eating” scale score. There is a moderation effect between self-esteem and the personality trait of extraversion on the “Uncontrolled Eating” subscale score. There is a moderation effect between self-esteem and the personality trait of conscientiousness on the “Uncontrolled Eating” scale score.

Table 1. Analysis and the moderation effect between self-esteem and the personality trait of conscientiousness in the effect on the “Cognitive Restraint of Eating” subscale score.

**Table:** 

		95% Confidence Interval			
	b	Lower	Upper	Z	p
C stens	-0.150	-0.220	-0.080	-4.214	< 0.001
Rsbrg	-0.001	-0.022	0.020	-0.097	0.923
C stens ٭ Rsbrg	0.010	0.000	0.019	2.040	0.041
Low (-1SD)	-0.221	-0.320	-0.122	-4.380	< 0.001
Average	-0.150	-0.220	-0.080	-4.200	< 0.001
High (+1SD)	-0.079	-0.176	0.018	-1.590	0.111
b – unstandardised regression coefficient, CI – confidence interval; ٭ moderation effect. *Note.* shows the effect of the predictor (conscientiousness) on the dependent variable (Cognitive Restraint of Eating) at different levels of the moderator (Rsbrg)

**Conclusions:**

Self-esteem was not a predictor of the occurrence of eating disorders while playing a moderating role in the relationship between certain personality traits and the occurrence of eating disorders. A higher level of neuroticism was identified as an important predictor of higher results for orthorexia, Cognitive Restraint of Eating, Uncontrolled Eating, and Emotional Eating. It was also demonstrated that the orthorexia risk index decreased with increased extraversion and openness to experience.

**Disclosure of Interest:**

None Declared

